# Prevalence and associated factors of proliferative diabetic retinopathy among adult diabetic patients in Northwest Ethiopia, 2023: A cross-sectional multicenter study

**DOI:** 10.1371/journal.pone.0303267

**Published:** 2024-05-10

**Authors:** Abebech Fikade Shumye, Mebratu Mulusew Tegegne, Biruk Lelisa Eticha, Matiyas Mamo Bekele, Asamere Tsegaw Woredekal, Lakew Asmare

**Affiliations:** 1 Department of Optometry, College of Medicine and Health Sciences, Comprehensive Specialized Hospital, University of Gondar, Gondar, Ethiopia; 2 Department of Ophthalmology, College of Medicine and Health Sciences, Comprehensive Specialized Hospital, University of Gondar, Gondar, Ethiopia; 3 Department of Epidemiology and Biostatistics, College of Medicine and Health Sciences, Wollo University, Dessie, Ethiopia; Debre Tabor University, ETHIOPIA

## Abstract

**Background:**

Proliferative diabetic retinopathy is one of the advanced complications of diabetic retinopathy. If left untreated, almost all eyes could lose a significant portion of their vision within four months. There is limited evidence regarding the magnitude of proliferative diabetic retinopathy and associated factors in the study setting and also in Ethiopia.

**Purpose:**

To determine the magnitude and associated factors of proliferative diabetic retinopathy among adult diabetic patients attending Specialized Comprehensive Hospital-Diabetic Care Clinics in Northwest Ethiopia, 2023.

**Methods:**

A multicenter, hospital-based, cross-sectional study was conducted on 1219 adult diabetic patients selected by systematic random sampling technique. Data were collected through an in-person interview and physical examination. The Statistical Package for Social Science Version 20 was used to analyze the data. Logistic regression methods were used to test the association between predisposing factors and proliferative diabetic retinopathy. The adjusted odds ratio with a 95% confidence interval was used to determine the strength of association.

**Results:**

The prevalence of proliferative diabetic retinopathy was 3.1% (95% CI: 2.10%-4.10%). Hypertension (AOR = 4.35 (95% CI: 1.87–10.12)), peripheral neuropathy (AOR = 3.87 (95% CI: 1.57–9.54)), nephropathy (AOR = 2.58 (95% CI: 1.13–5.87)), ≥10 years duration of diabetes mellitus (AOR = 5.30 (95% CI: 2.32–12.14)), insulin use (AOR = 3.07 (95% CI: 1.08–8.68)), and poor adherence to diabetes mellitus medications (AOR = 3.77 (95% CI: 1.64–8.64)) were confirmed to have statistically significant association with proliferative diabetic retinopathy.

**Conclusion:**

The prevalence of proliferative diabetic retinopathy among adult diabetic patients in the diabetes clinic was higher than the global study. Hypertension, peripheral neuropathy, nephropathy, ≥10 year’s duration of diabetic mellitus, insulin use and poor adherence to diabetes mellitus medications were among the factors significantly associated with proliferative diabetic retinopathy.

## Introduction

According to the World Health Organization, Diabetes Mellitus (DM) is defined as a chronic metabolic disease caused by elevated blood sugar levels in the blood vessels. Over time, it leads to damage to the heart, blood vessels, eyes, kidneys and nerves [1]. A microangiopathy caused by the long-term complications of diabetes mellitus is Diabetic Retinopathy (DR) [2].

Proliferative Diabetic Retinopathy (PDR) which is characterized by the development of abnormal new blood vessels at the optic nerve head or elsewhere in the retina is one of the serious complications of DR [3]. Uncontrolled high blood glucose levels in the blood vessels and untreated Non-Proliferative Diabetic Retinopathy (NPDR) play a vital role in the progression of PDR [3, 4]. It could be manifested as or end up with central and peripheral vision loss, vitro-retinal traction, preretinal hemorrhage, vitreous hemorrhage, tractional retinal detachment, and neovascular glaucoma [5].

The pathophysiology of PDR is associated with fibrosis, angiogenesis and inflammatory cells [6, 7]. Ocular neovascularization is facilitated by several genes, including erythropoietin, cytokines, and vascular endothelial growth factors, which are expressed by inflammatory cells through the processes of cytokinesis and chemokinesis [8].

According to 2019 study report by the International Diabetic Federation (IDF), the global prevalence of PDR was 1.40 [9]. The prevalence of PDR in eye clinics was found to be 33.4% in Sudan [10] and 50.5% in Jamaica [4] whereas the prevalence of PDR observed in diabetic clinics found in Ethiopia ranges from 0.7% to 16.7% [11–13].

Proliferative diabetic retinopathy is the main cause of blindness in DM patients. If left untreated, it has a high chance of progressing to high-risk PDR and an eye will lose a significant amount of its vision [5, 14]. The disease is associated with numerous social and economic burdens for the individual and the healthcare system [15]. Emotional stress, loss of productivity, dependence, stigmatization and social isolation are among the burdens. Those hinder the patient’s social activities and affect their quality of life [15]. Similarly, PDR causes both direct and indirect economic burdens through loss of productivity, increased medical costs and caregiver costs [15]. The cost of treatment and rescue from severe vision loss due to PDR in South Africa has been estimated to be $1,618 and $13,71313 respectively [16].

Type of DM [17, 18], hemoglobin [19–23], glycerol control [8, 19], duration of DM [18, 21–23], hypertension [23], insulin use [21, 23], creatinine level [23], lipoprotein density [17], hyperlipidemia [17] and proteinuria [19, 20] have been significantly associated with PDR development.

The International Council of Ophthalmology recommends diabetic patients have regular exercise, regular screening, timely referral to an ophthalmology center and routine eye examinations that help the endeavor to prevent the occurrence of PDR; to detect and treat it as early as possible and make patients aware of their condition [24, 25].

Knowing the magnitude and factors associated with PDR among adult diabetic patients is necessary to set appropriate interventions and administrative measures based on modifiable factors. However, there is limited evidence about the magnitude of PDR and associated factors in the study setting and Ethiopia as well.

Therefore, this study aimed to identify the prevalence of PDR and its associated factors among adult diabetic patients attending Comprehensive Specialized Hospital-Diabetic Care Clinics (CSH-DCCs) in Northwest Ethiopia to identify and fill the gap in terms of reference support for policymakers, program planners and decision makers that will improve early diagnosis and management of the problem.

## Methods and materials

### Study design, study area and period

A multicenter hospital-based cross-sectional study was conducted at the CSH-DCCs in Northwest Ethiopia, from May 8 to June 15, 2023. In Amhara Region, there are about 81 functional hospitals, 858 health centers and 3560 health posts [26]. Five of the hospitals named the University of Gondar, Debre Tabor, Felege Hiwot, Tibebe Ghion and Debre Markos are comprehensive specialized hospitals located in Northwest Ethiopia. Each comprehensive specialized hospitals has a separate adult diabetic clinics under the Department of Internal Medicine and is staffed by specialized internists, general practitioners, nurses and other medical professionals who provide service for both new and follow-up diabetic patients.

### Source population and study population

All adult diabetic patients attending at the CSH-DCCs in Northwest Ethiopia.

### Inclusion criteria

All adult diabetic patients with adequate visualization of the posterior segment in both eyes were included in the study.

### Exclusion criteria

Media opacity obscuring the visualization of the posterior segment in at least one eye including dense corneal opacity, mature cataract, and marked vitreous opacity developed by non-DM causes were excluded from this study. For the sake of getting reliable data patients who were seriously ill and unable to communicate were also excluded from this study.

### Operational definitions

#### Proliferative diabetic retinopathy

Defined as ‘Yes’ if any of the following were present: Neovascularization of the retina or optic nerve head, preretinal or vitreous hemorrhage, and tractional retinal detachment in at least one eye. ‘No’ if none of the above features were present in both eyes [27].

#### PDR classification

*Low-risk PDR*. Neovascularization of the disc or retina without vitreous or pre-retinal hemorrhage.

*High-risk PDR*. Neovascularization of the retina or disc with vitreous or pre-retinal hemorrhage.

*Advanced PDR*. High-risk PDR with tractional retinal detachment [27].

#### Hypertension

Defined as ‘Yes’ if the measured systolic and/or diastolic blood pressure of the diabetic participants were above 140/90 mm Hg during the data collection period or if the participants were taking antihypertensive medication. ‘No’ if both systolic and diastolic blood pressure was below 140/90 mm Hg during the data collection period and there is no known history of hypertension [28].

#### Poor glycemic control

Fasting blood glucose levels >130 mg/dl at the time of data collection [29].

#### Body Mass Index (BMI)

Based on the formula of weight in kilogram over height in meter square, the BMI was calculated to be classified as ‘underweight’ if the BMI was <18.50kg/m^2^; ‘normal’ if the BMI was between 18.50 and 24.99 kg/m^2^; ‘overweight’ if the BMI was between 25.00 and 29.99 kg/m^2^; and ‘obese’ if the BMI was ≥30 kg/m^2^ during the time of data collection [30].

#### Eye checkup practices

Participants who had undergone an eye examination within the past year were categorized as having good eye checkup practices, while those who had not undergone an eye examination within the past year were categorized as having poor eye checkup practices [31, 32].

#### Medication adherence

The study participants who answered below the median value [6] of the 7-point questions on treatment adherence were considered as having poor adherence to DM medication. However, the study participants who scored above or equal to the median [6] were classified as having good adherence to DM medication [33].

#### Adult

A person who was 18 years old and above [34].

### Sample size determination and sampling technique

The sample size was determined using a single population proportion formula by taking a 13.3% proportion of PDR obtained from a pilot test conducted on 30 subjects at Debre Tabor CSH-DCC. Furthermore, the sample size calculation considered a 5% significance level and a 2% margin of error.

The following formula was used;

$$n=\frac{\left(Z_{\alpha /2}{)}^{2}*P(1-P\right)}{d^{2}}=\frac{\left(1.96{)}^{2}*0.133(1-0.133\right)}{0.02^{2}}$$

n = 1107.4

Where

n = Sample size

Z = Z statistics for 95% level of confidence = 1.96

P = Estimated proportion of proliferative diabetic retinopathy

d = Margin of error = ±2%

After adding 10% non-response rate the final total sample size was set to be 1219.

**Fig 1:**
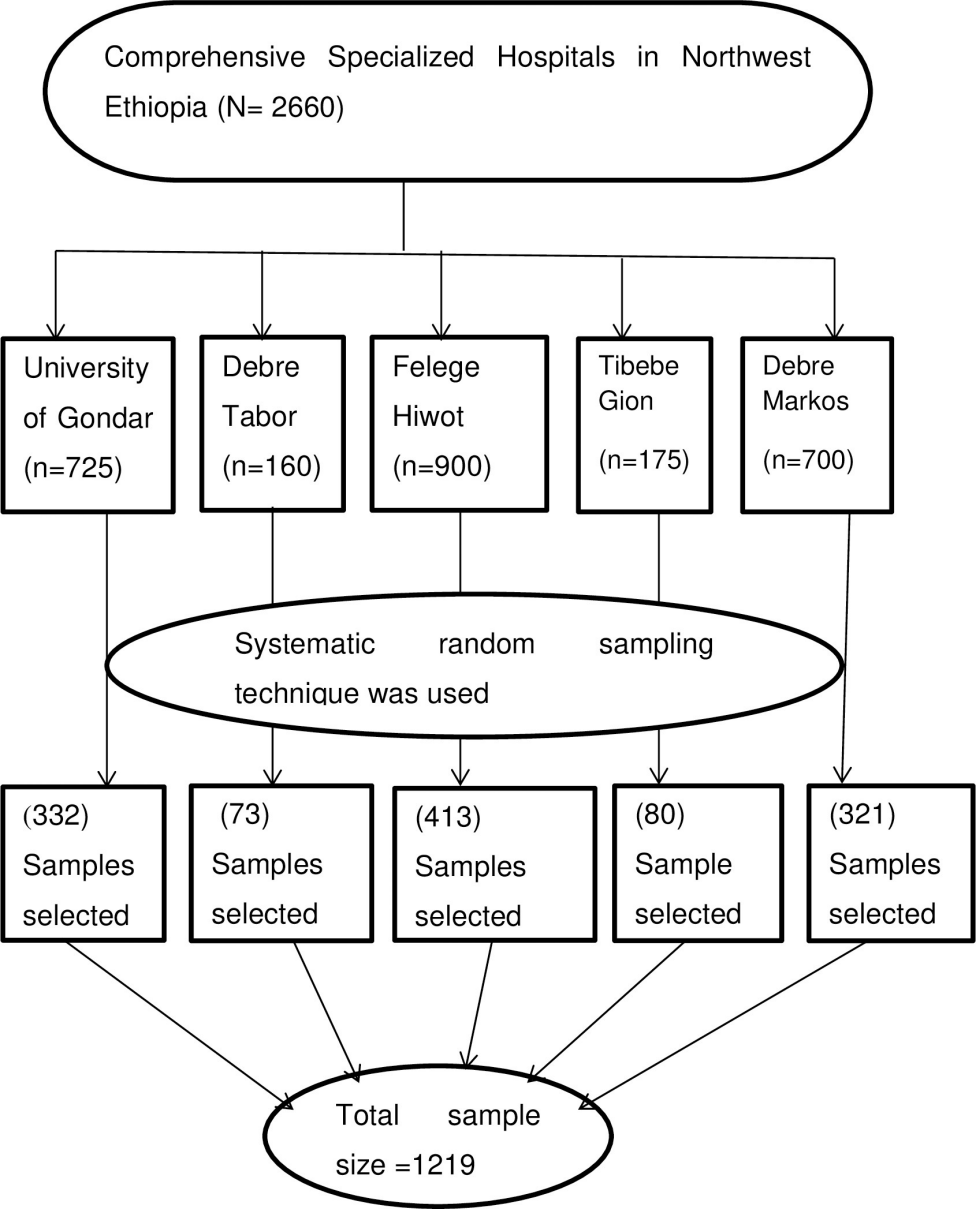
This flow diagram shows the sampling procedure and sampling technique of study participants.

The study participants were selected from five CSH-DCCs found in Northwest Ethiopia. Out of the total of 2660 expected average number of diabetic patients attending these CSH-DCCs 1219 study participants were selected using a systematic random sampling technique. Once we got the list of diabetic patients from the registration book proportional allocation was done for each CSH-DCCs. After allocation was done, the sampling interval was calculated and the value was 2. We used the lottery method to draw the 1st sample of the first 2 participants and continued with every other participant (Fig 1).

### Data collection tool and procedure

The data were imported to electronic device Kobo Toolbox version 2022.4.4. The semi-structured interview-based questionnaire includes sociodemographic, DM medication adherence, systemic and ocular comorbidity, and clinical characteristics related questions used to collect data.

Five trained optometrists and other five trained ophthalmologists collected the data through face-to-face interviews and physical examinations. The overall data collection was conducted under the close supervision of other five optometrists; 1 supervisor for each CSH-DCCs.

By reviewing related literature and considering the current clinical practice the questionnaire was developed in English and then translated to Amharic and later back to English by language experts to ensure the accuracy and reliability of data. Before the actual data collection, the Amharic version of the questionnaire was pretested on 5% of the sample size at Debark Hospital to check the reliability and quality. Based on the feedback we got proper correction was done on the questionnaire.

The socio-demographic characteristics and DM medication adherence comprised of seven questions were assessed by interviewing study subjects in a face-to-face manner. Systemic diseases such as (hypertension, nephropathy, and peripheral neuropathy), type of DM, blood pressure, and FBS, were recorded on the checklist by reviewing patients’ medical recording charts. These factors were diagnosed and measured by the physicians at the diabetic care clinic at the time of data collection. Height and weight were measured using a standiometer.

Once the habitual visual acuity was assessed using a reduced Snellen visual acuity chart at a distance of 3 meters, examining the anterior segment of the eyes using a slit-lamp biomicroscope became the next task of the ophthalmologist. Then, participants who were eligible for the fundus examination got a drop of 1% Tropicamide eye drop to both of their eyes and waited 20 minutes until mydriasis. As time went by eyes became dilated and ready for dilated fundus examination using slit-lamp biomicroscope and a +90.00 Diopter Volk lens. The presence and grade of PDR were recorded on the checklist after the complete ophthalmic examination by the ophthalmologist.

### Data processing and analysis

The collected data was exported to the Statistical Package Social Sciences (SPSS) version 20 for checking, cleaning and analysis. Descriptive statistics such as proportion, frequency, ratios and summary measures such as median and interquartile range were calculated. Chi-square was used to determine the relationship between dependent and independent variables. Bivariable logistic regression analysis was then performed to assess the crude association of the explanatory variables to the dependent variable. Variables with a p-value of less than 0.25 in the bivariable logistic regressions were fitted to the multivariable binary logistic regression model using the Enter method. In multivariable logistic regression, variables with a p-value of less than 0.05 at a 95% confidence interval were considered statistically significant associated factors for PDR. The Hosmer and Lemeshow test of goodness of test was used to assess the fit of the model, and the result of its p-value was 0.83.

### Ethical considerations

Ethical clearance was obtained from the University of Gondar, College of Medicine and Health Sciences, Comprehensive Specialized Hospital, School of Medicine, Ethical Review Committee. An official permission letter was obtained from the Departments of Internal Medicine in each Comprehensive Specialized Hospital. Before the start of data collection, verbal informed consent was obtained from study participants, in which they were informed of the purpose of the study and their right to withdraw or refuse participation at any time. The study was conducted under the principles of the Declaration of Helsinki.

Confidentiality of study participants was maintained by avoiding all personal identifiers from the data collection instruments and keeping the results locked and secure. If additional care and follow-up were required, participants were connected to the eye clinic.

## Results

### Socio-demographic characteristics of the study subjects

Of a total of 1219 study subjects involved 1134 with a response rate of 93% gave valid data regarding the research. The median age of the participants was 53 years with an interquartile range of 37 to 62 years (Table 1).

**Table 1:** Socio-demographic characteristics of adult diabetic patients attending Comprehensive Specialized Hospital-Diabetic Care Clinics in Northwest Ethiopia, 2023 (n = 1134).

Variable	Category	Frequency	Percent
Sex			
	Male	619	54.59%
	Female	515	45.41%
Age (in years)			
	18–27	119	10.49%
	28–37	168	14.82%
	38–47	291	25.66%
	48–57	385	33.95%
	>57	171	15.08%
Residence			
	Urban	800	70.55%
	Rural	334	29.45%
Educational status			
	No formal education	221	19.49%
	Primary education	386	34.04%
	Secondary education	336	29.63%
	College and above	191	16.84%
Occupational status			
	Government	187	16.49%
	Private	457	40.30%
	Housewife	215	18.96%
	Retired	174	15.34%
	Others*	101	8.91%
Marital status			
	Single	99	8.73%
	Married	879	77.51%
	Divorced	73	6.44%
	Widowed	83	7.32%
Health insurance			
	Yes	706	62.26%
	No	428	37.74%
Average family monthly income (in ETB)			
	≤ 2500	298	26.28%
	2501–4000	275	24.25%
	4001–6500	278	24.51%
	>6501	283	24.96%

Note: ETB—Ethiopian Birr, Other*—includes student, no job, and farmer, average family monthly income was classified by quartile

### Clinical characteristics of the study participants

The median fasting blood glucose level was 150 mg/dl, (IQR: 126–180 mg/dl). The median and IQR of systolic and diastolic blood pressure were 120 mm Hg (IQR: 120–140 mm Hg) and 80 mm Hg (IQR: 70–80 mm Hg), respectively. The median BMI was 23 kg/m^2^ (IQR: 20.70–26 kg/m^2^) (Table 2).

**Table 2:** Clinical characteristics of adult diabetic patients attending Comprehensive Specialized Hospital-Diabetic Care Clinics in Northwest Ethiopia, 2023 (n = 1134).

Variable	Category	Frequency	Percent
Type of DM			
	Type I	264	23.28%
	Type II	870	76.72%
Fasting blood sugar level			
	Poor control	836	73.72%
	Good control	298	26.28%
Systolic blood pressure			
	<140	973	85.80%
	≥140	161	14.20%
Diastolic blood pressure			
	<90	969	85.45%
	≥90	165	14.55%
Mode of treatment			
	Insulin	292	25.75%
	Tablets	610	53.79%
	Both	232	20.46%
BMI			
	Underweight	165	14.55%
	Normal	541	47.71%
	Overweight	267	23.54%
	Obesity	161	14.20%
Family history of DM			
	Yes	407	35.89%
	No	727	64.11%
Duration of DM (in year)			
	<10	926	81.66%
	≥10	208	18.34%
Eye checkup practices			
	Good practice	664	58.55%
	Poor practice	470	41.45%
Adherence to DM medication			
	Good	648	57.14%
	Poor	486	42.86%
Awareness towards DR			
	Yes	448	39.51%
	No	686	60.49%

Note: DM–Diabetes Mellitus, DR–Diabetic Retinopathy

### Adherence to diabetic medication

Approximately 43% of the study participants had poor adherence to DM medication (Table 3).

**Table 3:** Adherence to diabetic medication among adult diabetic patients attending Comprehensive Specialized Hospital, Diabetic Care Clinics in Northwest Ethiopia, 2023 (n = 1134).

Adherence questions	Category	Frequency	Percentage
Do you sometimes forget to take your treatment for diabetic mellitus?	Yes	961	84.70%
	No	173	15.30%
In the last two weeks was there any day when you did not take your diabetic medication?	Yes	890	78.50%
	No	244	21.50%
Have you ever stopped taking your medications or decreased the dose without first warning your doctor because you felt worse?	Yes	817	80.90%
	No	217	19.10%
When you travel or leave the house, do you sometimes forget to take your medication?	Yes	829	73.10%
	No	305	26.90%
Did you take your diabetic medication yesterday?	Yes	288	25.20%
	No	848	74.80%
When you feel your fasting blood sugar level is controlled, do you sometimes stop taking your medication?	Yes	807	71.2%
	No	327	28.80%
Have you ever felt distressed for strictly following your diabetic mellitus treatment?	Yes	708	62.40%
	No	426	37.60%

### Ocular and systemic comorbidities

About 14.37% (163) of the study subjects were suffering from a certain type of glaucoma. Besides hypertension was the most common type of systemic comorbidity with 30.95% (351) occurrence (Table 4).

**Table 4:** Ocular and systemic comorbidity characteristics of adult diabetic patients attending Comprehensive Specialized Hospital, Diabetic Care Clinics in Northwest Ethiopia, 2023 (n = 1134).

Comorbidity	Category	Frequency	Percentage
Age-related macular degeneration			
	No	1094	96.47%
	Yes	40	3.53%
Glaucoma			
	No	971	85.63%
	Yes	163	14.37%
Diabetic macular edema			
	No	1053	92.86%
	Yes	81	7.14%
Diabetic retinopathy			
	No	1011	89.15%
	Yes	123	10.85%
Hypertension			
	No	783	69.05%
	Yes	351	30.95%
Peripheral neuropathy			
	No	994	87.65%
	Yes	140	12.35%
Nephropathy			
	No	940	82.89%
	Yes	194	17.11%
Chronic foot ulcer			
	No	974	85.89%
	Yes	160	14.11%

### Prevalence of proliferative diabetic retinopathy

In this study, the prevalence of PDR was 3.10%, (95%CI: 2.10%–4.10%). Of this prevalence, 51.43% was low risk, 40% was high risk and 8.57% was advanced level.

### Clinical manifestation and treatment modality of PDR

**Fig 2:**
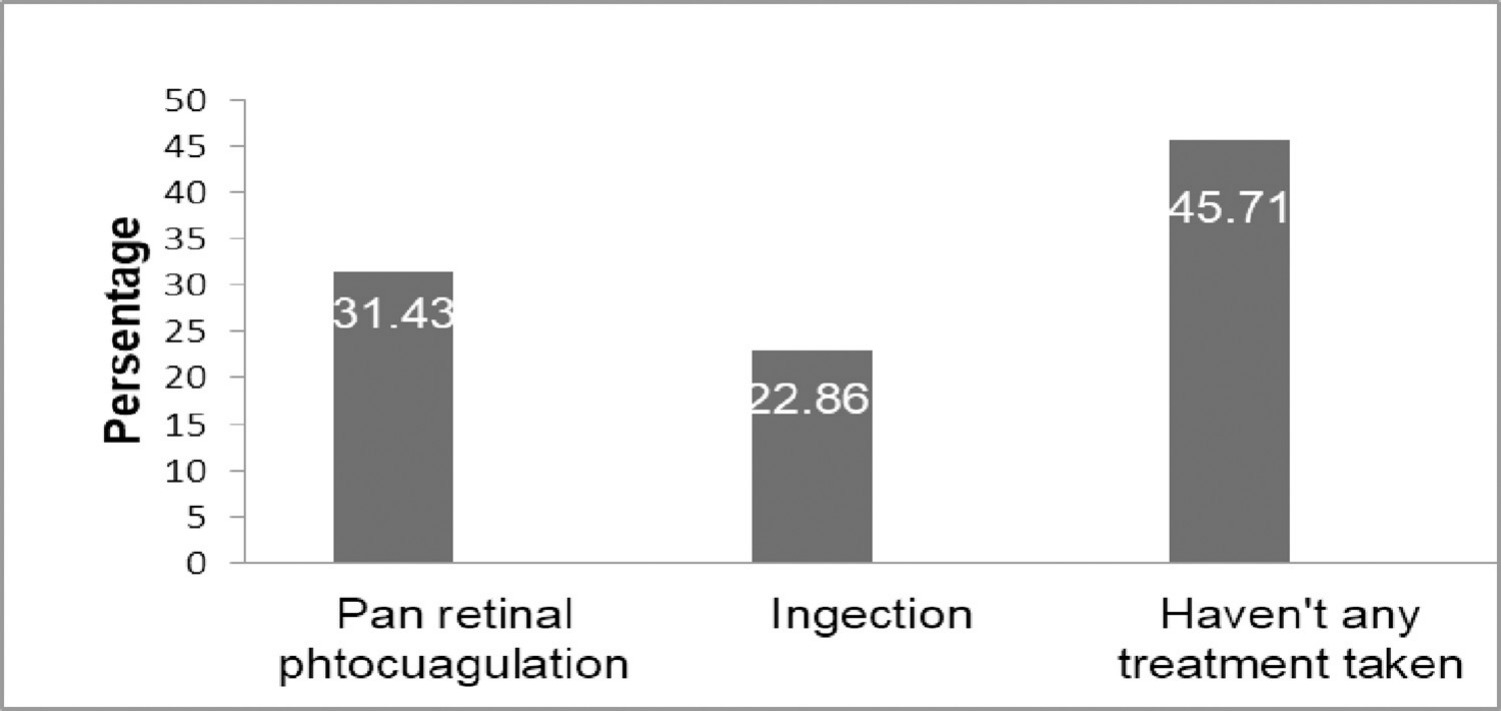
The treatment modality of PDR among adult diabetic patients in Northwest Ethiopia (n = 35).

Among the total PDR patients, the majority were manifested as new vessels on the optic disk 40.00% (14), new vessels in the retina 25.71% (9), vitreous hemorrhage 14.29% (5), preretinal hemorrhage 8.57% (3), and tractional retinal detachment 4 (11.43%). Nearly half of the PDR patients, 45.71% (16) had never started treatment to control the PDR they suffered from (Fig 2).

### Factors associated with proliferative diabetic retinopathy

All variables fulfilling the chi-square assumption were entered separately into the bivariable logistic regression model. Of these variables, glaucoma, duration of DM, treatment modality of DM, nephropathy, peripheral neuropathy, hypertension, eye checkup practices, awareness towards DR, and adherence to DM medication had a p-value of less than 0.25. Thus, these variables got the chance to undergo the multivariable logistic regression. In the multivariable logistic regression, hypertension, peripheral neuropathy, nephropathy, duration of ≥10 years with DM, insulin use and poor adherence to DM medication were found to be statistically significant factors for PDR.

Study participants living with hypertension were 4.35 times more likely to have PDR than participants without hypertension (AOR = 4.35, 95% CI: 1.87–10.12).

The odds of developing PDR was 3.87 times higher in participants having peripheral nephropathy as compared with participants not having (AOR = 3.87, 95% CI: 1.57–9.54). While, study participants who developed nephropathy had 2.58 times higher odds of having PDR as compared to participants who had no nephropathy (AOR = 2.58, 95% CI: 1.13–5.87).

Regarding the duration of DM, the odds of having PDR were 5.30 times higher in the study subjects living with DM for 10 years and above than those DM patients diagnosed within the past 10 years (AOR = 5.30, 95% CI 2.32–12.14).

This study unveiled that, the odds of having PDR among insulin users were 3.07 times higher as compared to participants who used the combination of therapy (AOR = 3.07, 95% CI 1.08–8.68). Finally not adhering to DM medication increases the odds of having PDR by 3.77 fold (AOR = 3.77, 95% CI 1.64–8.64) (Table 5).

**Table 5:** Factors associated with proliferative diabetic retinopathy among adult diabetic patients attending comprehensive specialized hospital-diabetic care clinic, Northwest Ethiopia, 2023 (n = 1134).

Variables	PDR		COR (95%CI)	AOR (95%CI)	P value
	Yes	No			
Glaucoma					
Yes	9	154	2.12(0.97–4.61)	1.87(0.75–4.64)	0.176
No	26	945	1.00	1.00	
Hypertension					
Yes	25	326	5.92(2.81–12.48)	4.35(1.87–10.12)	0.001
No	10	773	1.00	1.00	
Peripheral neuropathy					
Yes	10	130	2.98(2.17–6.29)	3.87(1.57–9.54)	0.003
No	25	969	1.00	1.00	
Nephropathy					
Yes	14	180	3.40(1.69–6.81)	2.58(1.13–5.87)	0.023
No	21	919	1.00	1.00	
Body mass index					
Underweight	5	160	1.00	1.00	
Normal	6	535	0.35(0.10–1.19)	0.52(0.14–1.91)	0.331
Overweight	12	255	1.50 (0.52–4.35)	1.47 (0.44–4.86)	0.523
Obesity	12	149	2.57 (0.88–7.49)	2.60 (0.78–8.72)	0.12
Duration of DM (in year)					
<10	13	913	1.00	1.00	
≥10	22	186	8.30 (4.1–16.78)	5.30(2.32–12.14)	0.001
Treatment of DM					
Insulin use	18	274	2.11(1.58–6.50)	3.07(1.08–8.68)	0.034
Tablets use	10	600	0.53(0.13–1.97)	0.49(0.16–1.45)	0.200
Both	7	225	1.00	1.00	
Eye checkup practice					
Good	11	653	1.00	1.00	
Poor	24	446	3.19(1.54–6.58)	1.41(0.60–3.31)	0.427
Adherence to DM medication					
Good	11	637	1.00	1.00	
Poor	24	462	3.00(1.30–6.36)	3.77(1.64–8.64)	0.002
Awareness towards DR					
Yes	22	426	2.67(1.33–5.36)	1.28(0.56–2.89)	0.553
No	13	673	1.00	1.00	

Note: AOR—Adjusted Odds Ration, COR—Crude Odds Ration, CI—Confidence Interval, DM—Diabetes Mellitus, DR—Diabetic Retinopathy, PDR–Proliferative Diabetic Retinopathy

## Discussion

In this study, the prevalence of proliferative diabetic retinopathy was 3.10%, (95% CI: 2.10%–4.10%). This result was consistent with a couple of studies conducted in the United States 2.4% [35] and 2.3% [36], and a certain study done in Gondar, Ethiopia 3.6% [30].

The prevalence of PDR found in this study was lower than in previous studies conducted in Bosnia and Herzegovina 33.73% [37] and Malawi 4.8% [13]. This discrepancy could be due to the differences in the study settings. This study was conducted among all adult DM patients at the diabetic clinic. In contrast, the study in Bosnia and Herzegovina was conducted among adult DM patients attending eye clinics. The patients who attended eye clinics usually had visual complaints that could be associated with PDR. Those who did not attend eye clinics generally had no visual complaints. Thus, the prevalence of PDR is higher in diabetics who attend eye clinics than in the general diabetic population [30].

Similarly, the finding of this study was also lower than that of the studies in Jamaica 50.5% % [4] and Sudan 33.4% [10]. This difference could be due to the variation in the unit of analysis. In this study, individuals were analyzed, while in the study in Jamaica, eyes were analyzed. Thus, in the Jamaican study, bilateral PDR was counted as two separate outcomes, whereas in this study it was only one outcome. Religious and cultural differences could also contribute to the differences. In Jamaica, diabetics see their disease as a punishment from God and do not take the necessary medication. This might lead to higher rates of PDR due to uncontrolled blood glucose levels [38]. Furthermore, these studies were only conducted among adult DM patients attending eye clinics [30]. Therefore this finding suggests that increasing community awareness regarding medication adherence will reduce the severity of PDR.

The result of this study was also lower than the studies conducted in China 12.8% [39], Lebanon 8.9% [40] and Addis Ababa, Ethiopia 16.7% [11]. This could be due to the variation in the study population and duration of the study. Studies done in Addis Ababa and Lebanon were conducted among type II DM patients only. Furthermore, the prevalence of PDR in Addis Ababa was reported only among those who developed diabetic retinopathy. Since PDR is more common among DR patients and the denominator does not include all DM patients, its magnitude will be overestimated. The other possible justification might be the study period difference. The study done in China was a period prevalence study. Since old and new cases were included in period prevalence, it is expected to be relatively higher than this point prevalence study.

On the other hand, the result of the current study was higher than a study conducted in Gondar 0.7% [12]. This might be due to differences in study size. The current study was conducted in a multicenter setting where the probability of getting PDR was high. In contrast, a study conducted in Gondar was conducted in a single center.

Living with DM for more than 10 years increases the odds of developing PDR by five times as compared to DM confirmed within the past 10 years (AOR = 5.30, 95% CI 2.32–12.14). With increasing duration, the pancreatic beta cells may become more resistant to the DM treatment. As a result, glucose levels in the blood vessels will increase to exacerbate the development of PDR [41]. This result was in line with the study conducted in Malaysia [17]. This agreement could be due to similar characteristics of the study participants. For example, the majority of participants in both of the studies were confirmed to have DM within the past 10 years, had bilateral PDR, many of them were taking oral hypoglycemic therapy and the age of the study participants was over 18 years.

This finding was also supported by studies done in China (AOR = 1.75) [18] and United States (AOR = 1.62) [21], (AOR = 22.00) [23]. Even if the strength of association was slightly different, those were supportive clues for the positive association of ≥10 years duration of DM with PDR. This strength of association difference might be due to genetic variation. A study conducted in Chinese reported that the vascular endothelial growth factors gene was not correlated with PDR [42].

This result shows that as the duration of DM increases, there is a higher probability of developing PDR. This finding suggests a paramount merit of linking or recommending DM patients to the eye care clinic as soon as possible. The ocular follow-up by itself could give us clues regarding controlling the DM status which supports each level of prevention of blindness related to PDR.

The odds of PDR in the participants who had peripheral neuropathy were four times higher than those who had not peripheral neuropathy (AOR = 3.87, 95% CI: 1.57–9.54). The result of this study was in agreement with a study done in Portugal (AOR = 6.76) [22]. This positive association could be due to the loss of peripheral nerve fibers. As a result, blood supply-induced ischemia leads to motor, sensory, and autonomic fiber dysfunction. The retina is going to form aberrant new blood vessels to get sufficient oxygen to overcome this DM-related ischemia. This aberrant vessel exacerbates the PDR development [22].

This finding was also supported by a study conducted in Malaysia (AOR = 14.23) [17]. Even though the probability of PDR among those who had peripheral neuropathy in the Malaysian study was slightly higher than in this study, it is supportive evidence of this result. This slight difference in the strength of association might be due to variations in the diagnostic criteria of peripheral neuropathy. Peripheral neuropathy was diagnosed based on the symptoms of the participants in the Malaysian study, while it was diagnosed clinically by the physician in this study.

The odds of PDR occurrence among subjects who developed nephropathy were three times higher than those participants without nephropathy (AOR = 2.58, 95% CI: 1.13–5.87). This figure was consistent with a report from United States (AOR = 1.29) [19]. The possible reason for this similarity could be disease metabolism. The increment of kidney creatinine or proteinuria leads to microvascular abnormalities [43]. This causes retinal tiny vessels to be damaged, which has serious consequences like increasing retinal venular diameter and leakage of vessels that might be more likely to have caused the development of PDR [20].

In addition, this finding was also supported by the study held in Malaysia (AOR = 10.23) [17]. This strength of association variation might have occurred due to the difference in the study population. The Malaysian study enrolled type II DM patients who are more exposed to developing late complications of DM that might inflate the strength of association but in this study both type I and II DM patients were included [44].

The result of this study showed that participants with hypertension were four times more likely to develop PDR than participants without hypertension (AOR = 4.35; 95% CI: 1.87–10.12). Hypertension facilitates PDR development through increased vascular endothelial growth factor production and hemodynamic changes. The 40% increase in mean arterial blood pressure has also the ability to impair retinal autoregulation processes leading to irreversible retinal microvascular structural change that causes PDR [45]. Therefore, this finding implies the advantage of preventing hypertension to reduce the occurrence of PDR. This finding was supported by a study in the United States (AOR = 1.64) [23]. Similarity of the study population characteristics could be mentioned as the possible cause of overlapping. Both studies enrolled both types of DM patients with similar proportions.

Using insulin as a choice of therapy increases the ability to develop PDR three times as compared to treating DM by a combination of insulin and tablet (AOR = 3.07, 95% CI 1.08–8.68). In terms of glycemic control, combined treatment is more effective than insulin alone [46]. The other probable justification was that the function of insulin is to regulate the metabolic functions of insulin-responsive tissues like the liver, adipose tissue, and skeletal muscle. But here in the retina insulin stimulates retinal neuronal development, differentiation, growth, and survival which causes cell dysfunction and death. This results in a high potential to develop PDR [47]. In addition, a higher sensitivity of insulin to environmental factors might make it partially or completely impotent, making insulin less efficient in controlling blood sugar levels in developing nations like Ethiopia. This might also be another possible reason for the observed positive association [48]. Keeping insulin in a room having proper temperature may help control DM, and PDR as well. Especially for those patients having poor adherence to their medication considering the combination of therapy may help more.

This finding was parallel with a couple of reports from the United States (AOR = 6.65) [21] and (AOR = 1.85) [24]. The similarity of the study population could be a possible explanation for being parallel. The possible reason for this positive relationship could be the therapeutic effect.

According to this study, participants who poorly adhered to their DM medication had four times higher odds of having PDR as compared to those of well adhered (AOR = 3.77, 95% CI 1.64–8.64). The possible reason might be type of DM, and treatment of DM. Asymptomatic nature of type II DM, older age, and comorbid conditions could be mentioned as the possible reasons that hinder compliance of type II DM patients to their medication [49, 50]. Insulin alone might have poor compliance due to inappropriate placement, perception and treatment complexity [49, 51]. Spiritual well-being was inversely correlated with treatment regimen adherence to DM patients. While directly correlated with hope and life happiness [4].

The results of this study revealed that poor adherence to DM medication results in more complications due to an uncontrolled blood sugar level. One of the consequences is microvascular abnormalities that aggravate the development of PDR [4]. Therefore, good medication adherence is crucial to prevent the development of PDR.

Overall, this study was more representative of the general diabetic population in Northwest Ethiopia due to its multicenter study design. However, there were some limitations in our study. The first was that, unlike other previously conducted studies in developed countries, we did not use a fundus camera for the diagnosis of PDR. Secondly, blood glucose control was assessed by fasting blood glucose level as there were no facilities to measure HbA1c at the study site. In addition, because income was not recorded using the standard wealth index, personal estimation errors may have occurred in the collection of income data.

## Conclusion

The prevalence of PDR among adult diabetic patients was relatively higher than the global one. Hypertension, duration of DM ≥10 years, peripheral neuropathy, nephropathy, insulin use and poor adherence to DM medication were significantly associated factors with PDR. In this study, around half of the PDR study participants had DM never treated so far. Approximately forty-three percent of the study subjects had poor adherence to DM medication, and two-thirds of the study participants had poor glycemic control.

## Recommendation

To health-care professionals, it is recommended to give counseling or follow closely whether the patients adhere to their medication. In addition, regular eye examinations at diabetes clinics and established referral linkage to eye clinics are recommended to prevent, detect, and treat PDR early. Furthermore, considering combination of therapies, especially for those having poorly controlled blood glucose levels is recommended to delay the occurrence of PDR. It would be better for the researchers if further studies are conducted at a national level using an advanced instrument (fundus camera) for better detection of PDR.

## Supporting information

S1 Data (CSV)

## Abbreviations

BMI: Body Mass Index
BP: Blood Pressure
DM: Diabetic Mellitus
DR: Diabetic Retinopathy
NVD: New Vessel on Disk
NVE: New Vessel Elsewhere
PDR: Proliferative Diabetic Retinopathy
PRH: Pre Retinal Hemorrhage
VH: Vitreous Hemorrhage
